# Bibliographical

**Published:** 1862-06

**Authors:** 


Bibliographical.—Through the kindness of Dr. Allport, we are in possession of a number of old volumes upon dental science, some of them extending back about one hundred years. We propose occasionally to give some extracts from these volumes, with a view of giving the ideas entertained at that time upon the various points of dental practice. Some of these are very singular, and others show that there were correct ideas entertained upon dental practice, even at that period.

The following consists of some extracts from a work entitled, An Essay on the Formation, Structure and Use of the Teeth, with a Supplement. By Mayer Lewis, Operator for the teeth in Oxford. London, 1772.

In regard to the scope of the work, the author says:

My design is merely to give a plain and brief account of the teeth, the complaints they are subject to, and the methods of cure; omitting nothing, I hope, which is necessary to be known, and avoiding all obscurities of style and meaning.

The following is his description of the teeth:

The teeth are hard substances of bone and enamel growing in the cavities of the upper and lower jaws. These cavities anatomists call the *alveoli*, which have circular processes embracing the body of the tooth with great closeness, and thereby are to be considered as principal supporters of the teeth.

The *shape* of the teeth being within the immediate extent of every one's own knowledge, I shall not here make mention of it. Their *number* is uncertain; though I believe they rarely exceed thirty. Mr. Hunter says, that he once saw only twenty-seven, but never more than thirty-two; and makes thereupon this very sensible remark, "That where the number is less than thirty-two, the deficiency is in the last grinder." This is, however, (I mean the number of teeth in a full subject) a point of mere curiosity; since during the course of my practice, I never could discover any difference in the formation or structure of them, whether more or less in number; but they all equally as well perform their usual functions, and answer the purposes for which they are designed. The number is not certain, I think, for this reason, viz., that if it were, when the jaw bone of a man is enlarged, and grows in proportion to the increase of his age, we do not find any difference in the *alveolar spaces*, either with regard to their number or extension, which would otherwise undoubtedly happen in the case between a man, who in his perfect state, had thirty-two teeth, and of another, who had only thirty.

The following upon the formation and irruption of the teeth embraces very nearly correct ideas:

The contents of the *alveoli*, or cavities in the jaw, in which the teeth grow, are, at almost the earliest state of infancy, some little soft pulpy substances, which are attached to the bottom of the sockets by a nervous filament, whose vessels diffuse themselves through the pulpy substances and the thin membrane that covers them. Nourishment is hereby conveyed to these pulpy substances, which, as the child increases in age, begin gradually to ossify, and advance higher and higher from the sockets. The tooth is yet imperfectly formed, not having gained any degree of strength and rigidity. By degrees they begin to press against the gums, which, by constant attrition are inflamed and affected with redness and swelling, and occasion great pain and uneasiness to the infant. What is called *the cutting of the teeth* now begins, which is a violent effort of nature to pierce through the confinement of the gums, where the teeth have undergone the state of formation and ossification, and to appear above the sockets. As this operation of nature is attended with great and continued pain to the child, and frequently not finished without the hazard of much danger, it has been a matter of surprise to some gentlemen of eminence in surgery, that a new and more expeditious method has not been introduced to assist the cutting of the teeth, and that the efforts and endeavors of nature have not been, as in almost all other cases, promoted by the assistance of art. Those gentlemen have thought, and with great appearance of reason and good sense, that no danger or inconvenience would attend the opening of the gums at the particular time of the first appearance of dentition, with an instrument, and thereby, by making a way for the teeth to raise themselves from the sockets to prevent the slow advances of nature. The instrument certainly must be managed with a nice and skillful hand, or it may, by penetrating too deep, do much harm and damage to the tooth itself, not yet sufficiently hardened. I can not suggest to myself any plausible objection to the propriety and expediency of this plan; however, I willingly submit this opinion to the judgments of those gentlemen, who claim a superior knowledge in this point, from their attendance upon children in their infancy; my practice having been chiefly confined to the care of the teeth in their full grown and perfect state.

The ideas upon the functions of the teeth are good. After having spoken of mastication and speech, he says:

A collateral advantage arising from the care and preservation of the teeth is the grace and symmetry they give to the countenance, keeping the complexion in a state of youthful bloom, and preventing all wrinkles about the face, which the depression of the jaws, in consequence of the loss of our teeth, would inevitably occasion. It is, I am told by my own and other people's observations, a prevailing fashion in the present age to keep the face and the complexion as delicate and smooth as art can make them: (I speak not here of our English ladies, whose care in this particular is laudable, graceful, and characteristic of that amiable part of the creation), but, without meaning any offense to that part of mankind, whom the uncouth rusticity and unpoliteness of some amongst us think they disgrace by giving them the appellation of *Beaus.* I beg leave to remind the younger part of my readers, that though they may possibly take *too much* pains in adorning their persons, and thereby incur the charge of effeminacy and the want of a decent and proper manliness, yet I think they may oftentimes take *too little* care in this particular; and none can be attended with more disagreeable and offensive sensations, both to themselves and their friends, than the neglect of the teeth. Excuses may be made frequently for any neglect of *dress*—slovenly apparel—or unpowdered curls, which may be occasioned by a want of time to undergo the various processes of dressing, or the avocations of business. But no excuses can be made or taken for a dirty and foul set of teeth, which argue an habitual neglect of them, and either a wilful or (what is just as bad in this case) an unintentional omission of the employment of cleaning them; which employment, when rightly understood, and frequently practiced, is attended with no length of time, nor the least trouble.

Of the diseases of the teeth he says:

The disorders to which the teeth are subject, either simply from the neglect of them, or from an ill habit and state of body, are numerous. Suffice it here to mention a few, a perfect knowledge of which will, I am persuaded, give weight to my opinion and advice, relative to the care of them. The tooth-ache is a pain which, as it arises from various causes, I suppose there are few amongst us who have at all times been totally exempt from. The pain and anguish are more violent and excruciating than the visitation of diseases to other parts of the body. Shakspeare, the immortal poet of the English nation, says:

——"There never was philosopher yet.
Who could endure the *tooth-ache* patiently;"

Meaning certainly thereby, that with whatever great fortitude and philosophical patience other disorders might be borne and combated with, the pain of the tooth-ache surpassed all human resolution and courage. The anguish is frequently so insupportable that it forces us to use violent means to obtain some kind of temporary ease, which means occasion, not a long time after, a greater and more afflicting return of the disorder. Such are, I think, unnecessary extraction of the teeth, which, according to some modern operators, is the universal method of cure; without considering that the pain may be oftentimes alleviated by gentle methods, and the tooth preserved entire. It frequently happens that the pain is severely felt in sound teeth, owing to ulcers and excrescences on the gums, sudden colds, from sympathy with the neighboring affected parts, or other causes. Can the extraction of those sound teeth be therefore in such cases deemed necessary, or even expedient? Common experience and sense show the contrary. We can not perform the operation without increasing the evil which it is intended to diminish or remove. By uncovering the roots, and baring the sockets of the neighboring teeth, we make them loose and susceptible of pain, and cause frequent inflammations in their nerves and vascular parts. I have known not a few instances where the extraction of one of the *molares* or grinders has considerably weakened the jaw, though performed in a skillful manner, and by a violent distention of the muscles of the jaw, brought on a great soreness and inflammation of them. I am, however, a great friend to extraction when the circumstances of the case positively demand it. Another method no less injudicious of expelling the pain, and gaining a temporary relief, is the filling the mouth with acrid and hot spices and substances, and burning spirituous liquors, which deaden all sensation for awhile, but when their influence is at an end, the pain returns with redoubled strength and severity; exclusive of the harm which such applications certainly do the gums by disposing them to inflammations, and occasioning sorenesses and external swellings.

The tartar of the teeth is another cause of their destruction, which is a yellowish substance formed on the teeth, which hardens in time, and forms a complete incrustation over them; deforming and discoloring them, and by secretly and imperceptibly insinuating itself in the *interstices* of the teeth, opens too great a passage between them, prevents the growth of the gums by an inconvenient pressure of them, and is productive of a numerous train of disorders. This tartar, however, when *early discovered*, is very easily removed, being at first of a soft pulpy nature, and may be rubbed off the teeth by washing the mouth with clean water, and afterwards gently rubbing the teeth with a soft spongy brush, or by using any common tooth powder. When, however, it has formed an incrustation too hard to be rubbed off in that manner, having acquired a strong adhesion to the teeth, the operator's instrument must be used, but great care is necessary to be taken in the use of it, so as not to damage the enamel. Some kind of an inflammation in the gums generally attends this operation, which inflammation may, however, be reduced with gentle means.

The tooth-ache is frequently communicated by *sympathy*, from one tooth to another. This may seem surprising, but I find all writers on the subject I have consulted, agree in the opinion, though without assigning any cause for so extraordinary a phenomenon. We must not, however, carry our notions of sympathetic affections so far as I remember one writer of my own country has done. I mean one *Durlach Cosel*, who was a German, and lived, I think, in the year 1558 under Ferdinand the First. In a treatise of his writing upon surgery, (which I remember to have seen in manuscript in Leipsic) he positively affirms, that sympathy may be communicated *by contact* to other animals; and mentions some instances where he has known the tooth-ache transferred from a man to a cat, by applying the man's cheek for a considerable time together, to that of the cat. The tooth-ache left the man, and the animal was seized with the violence of the pain, as was supposed, by the agony she seemed to labor under, and the squalling she made. This will, I make no doubt, be looked upon by all serious and sensible people as a foolish notion, and not founded in fact; and indeed I am greatly surprised that that writer should so warmly insist on the reality of it, as he was undoubtedly, by several works which he left behind him, a man of great wisdom and learning. * * * * * *

When the scurvy, or other putrid humor, lies preying upon the roots or fangs of the teeth, removed beyond the reach of those instruments that can extract it from the gums by lancing them; when it destroys the nerves, and corrupts the foundation of the tooth; in such cases, I must deem extraction necessary, and that it is so, will incontestibly appear, since we find the root or fang of the tooth quite covered with the corrupted matter, when we have pulled it out; which corrupted matter there is no possibility of reaching with our instruments, or expelling by any other means than extirpation, as it lies so close and concealed. Were the tooth to continue in the head, the corrupted matter would eat the tooth gradually upwards, and occasion a putrid decay of the gums. Stumps or portions of the roots of teeth which have either been broken off, or injudiciously extracted, ought certainly to be drawn. There is not much pain in this operation, when skillfully performed, in a comparative sense with regard to *whole* teeth being extracted. Mr. Beardmore's opinion is, that "when any tooth-ache arises from a portion of the root left behind, if the patient is unwilling to try this second operation, the pain may be sometimes removed by burning the nerve, or by applying a very small bit of lint dipped in essential oil of cinnamon, over the hollow part of the stump, or by introducing a bit of paste made of opium, camphor, and essential oil of peppermint." But with all possible deference to his opinion, I should certainly prefer the extraction of the stump, and recommend it in such strong terms to the patient as would, I think, induce him to submit to the operation. Palliative applications may give ease, though Mr. Beardmore seems himself to own the case to be uncertain, by making use of the word "*sometimes;*" but I think the pain will return in a short time, or the gums will grow over these stumps, and being constantly wounded by them, will be exposed to frequent inflammations and extremities of pain. I am happy in having Mr. Beardmore's authority to sanction my opinion, that "The stump or root of a tooth is at all times easily taken out, unless it grows to the jaw bone, which is a very rare case; and nothing is more erroneous than the popular notion that stumps are very difficult to be removed, and that digging and punching (as they call it) are absolutely necessary."

On filling he has the following remarks, which shows that the practice in reference to filling was rather in advance of what it is ordinarily supposed to have been. He speaks of filling the "cavity in which the nerve formerly lodged." The method of attachment is rather peculiar:

Stopping the teeth, or filling them with gold or lead, is a very useful practice, and attended with no inconveniences. This is necessary when the internal part of the tooth is destroyed, or wasted away, and something must be put in the cavity, to prevent the air from piercing to the fang, or root of it, and occasioning the tooth-ache, or a further decay; as also for hindering loose bits of our food, etc., from lodging in the said cavity. The cavity then, in which the nerve formerly lodged, must be well filled up with gold or lead, and, if occasion be, may be fastened by a wire or thread to the neighboring teeth; however, after it has been well pressed down in the hole, there will be no danger of its falling out.

He expresses a high appreciation of artificial teeth, and speaks well of their efficiency. The manner of inserting them he does not describe very clearly, or we do not fully understand it. It certainly differed much from that employed now.

When there is a real necessity of extracting teeth, or they grow loose from any venereal or putrid disorder in the blood, I am a great friend to the practice of supplying their places with artificial teeth. After a little use, they equally as well perform the different offices of real teeth, in mastication, in speech, and in appearance. Indeed to such a pitch of greatness has the art and ingenuity of modern times arrived, that operators can make them, and fit them in the sockets in such a manner as not to be discernible from our natural teeth. After washing the mouth constantly for some time afterwards with some astringent liquors, we find that the gums will closely adhere to these artificial teeth. I have frequently made and set whole rows of artificial teeth, which have looked very ornamental, and have given an healthy air to the countenance. The general objection seems to be that they do not fit firm and easy in the sockets, but are apt to fall out. This objection, I flatter myself, in the course of my practice as a dentist, I can wholly obviate; for without meaning or wishing to be my own panegyrist, or desirous of arrogating any credit to extravagant professions which some modern operators for the teeth among us do, I can safely pronounce that this branch of the profession has employed greatly my time and attention, and I have, in consequence of that attention, discovered a method of setting and fixing them almost immovably in the sockets, without the great number of wires and ligatures which are commonly used.

